# Detection of Kernel-Level Spoilage Adulteration in Dried Goji Berries Using Zero-Shot Learning and Computer Vision

**DOI:** 10.3390/foods15162869

**Published:** 2026-08-17

**Authors:** Ruobin Huang, Yuanning Zhai, Baiwei Sun, Osama Elsherbiny, Lei Zhou, Yiying Zhao

**Affiliations:** 1 College of Mechanical and Electronic Engineering, Nanjing Forestry University, Nanjing 210037, Chinaynzhai@njfu.edu.cn (Y.Z.); baiweisun@njfu.edu.cn (B.S.); 2 Agricultural Engineering Department, Faculty of Agriculture, Mansoura University, Mansoura 35516, Egypt; osama_algazeery@mans.edu.eg; 3 Institute of Digital Agriculture, Zhejiang Academy of Agricultural Sciences, Hangzhou 310021, China

**Keywords:** food fraud, deep learning, berry localization, freshness grading, food inspection

## Abstract

Hidden adulteration of stale berries in dried goji berry batches is difficult to detect by manual inspection or batch-level quality assessment. This study developed a high-throughput method for kernel-level spoilage adulteration quantification in dried goji berries. It addressed three practical challenges in the image processing of densely arranged dried-fruits, including scalable label generation for deep learning segmentation without pixel-level manual annotation, separation of densely touching small berries, and full-size quality level distribution map reconstruction. SAM-assisted pseudo-label generation combined with multi-scale image cropping was used to overcome the limitation of manual pixel-level annotation, while YOLO-based instance segmentation was further employed for efficient berry localization in dense scenes. The freshness labels of segmented single berries were assigned by a statistical RGB-HSV grading rule. Specifically, adaptive multi-scale image cropping for segmentation was applied to improve local separability of berries under dense adhesion and occlusion conditions. The crop-level segmentation and grading outputs were subsequently reconstructed into the original image coordinate system to generate complete quality distribution maps. Results showed that YOLO models trained based on the pseudo-labels achieved a precision of 0.953, a recall of 0.951, an mAP_50_ of 0.960, and an mAP_50-95_ of 0.846. The full-size grading map reconstruction method produced a mean duplicate-suppression rate of 4.31%. In the full freshness-grading test dataset, 4850 berries were detected, including 449 stale berries. The mean absolute counting error was 1.61%. The proposed framework reduces manual annotation requirements while enabling berry-level freshness classification and quantitative stale-berry proportion estimation, providing objective information for dried fruit quality screening and adulteration control.

## 1. Introduction

Goji berry is a high-value crop widely used in foods, nutraceuticals, and health products. Its market value depends strongly on appearance traits, including color, gloss, integrity, and apparent freshness [1,2]. These traits influence consumer acceptance, commercial grading, product pricing, and brand reputation. Among them, freshness-related visual characteristics are especially important because they directly reflect perceived product quality and commercial desirability. At the final packaging stage, stale berries are sometimes mixed with fresh products, either unintentionally during handling or deliberately for economic gain. Unlike obvious defects such as breakage or mold contamination, stale berries usually retain their overall morphology and therefore can be easily concealed within high-quality batches. Their deterioration is mainly reflected by reduced gloss, color changes, and loss of apparent freshness rather than severe structural damage. As a result, even a small number of stale berries may substantially affect the perceived quality of a batch while remaining difficult to detect through routine visual inspection. This hidden mixing poses a challenge to quality grading and market supervision of dried goji berry products [3,4]. Therefore, methods capable of identifying individual stale berries and quantifying their proportion within a batch are needed to support more precise quality assessment and quality-control decisions.

Current quality assessment of dried goji berries still relies mainly on manual inspection and batch-level analytical methods, such as chemical tests and spectroscopy [5,6,7,8]. Manual inspection is subjective, slow, and hard to standardize. Instrument-based methods often need sample preparation, special equipment, and higher cost. More importantly, these methods usually give only batch-level results. They can-not show where the stale berries are or how many are mixed in the batch [9]. Therefore, individual-berry-level analysis is required for more precise quality assessment and hidden stale-berry detection. Computer vision has been widely used in food and agricultural quality inspection [10,11,12,13]. However, most previous studies focused on image-level classification, defect detection, or maturity grading [14,15,16]. These approaches mainly analyze overall image characteristics or predefined defects and are generally insufficient for individual-berry-level localization and quantitative estimation of stale berries within a batch, especially under dense touching and overlapping conditions. For dried goji berries, individual-berry analysis is particularly important because stale berries usually retain similar morphology to normal berries and mainly differ in subtle visual characteristics. Many image-based studies rely on manually separated samples, where individual berries are placed apart to facilitate berry identification and quality evaluation. Although this arrangement improves berry visibility, it requires considerable labor for precise sample preparation and can only process a limited number of samples, making it unsuitable for high-throughput inspection.

In practical applications, large numbers of particle-shaped food samples are usually analyzed simultaneously, where the target objects are often densely packed and exhibit frequent contact or overlap with neighboring particles. Recent advances in computer vision, deep learning, and vision foundation models have provided new opportunities for non-destructive inspection of agricultural products [17]. Object detection [18,19], instance segmentation [20,21,22,23,24], and image-based analysis methods have shown great potential in automated grading and quality evaluation [25,26]. However, conventional AI models require task-specific training based on manually annotated datasets, where target objects need to be labeled to guide model learning. For densely arranged particle-shaped food samples, generating accurate pixel-level or instance-level annotations is highly time-consuming and labor-intensive, which limits the scalability and practical deployment of AI-based inspection systems.

To reduce the dependence on task-specific annotation, zero-shot learning has emerged as a promising approach that enables models to generalize to unseen categories or domains without additional task-specific labeled training data. The Segment Anything Model (SAM), trained on large-scale general vision datasets, has demonstrated strong transferability to previously unseen objects and application domains [27]. This characteristic provides the possibility of transferring general visual knowledge to specialized fields such as food inspection. However, directly applying SAM to densely arranged dried goji berries remains challenging because touching and overlapping berries may lead to inaccurate instance separation or unreliable mask generation under highly crowded conditions. To address these challenges, this study proposes a SAM-assisted framework that combines adaptive multi-scale image cropping and pseudo-label generation. The generated pseudo-labels are further used to train a YOLO-based instance-segmentation model for efficient berry localization, combined with RGB-HSV-based freshness grading for berry-level classification and batch-level stale-berry proportion estimation. These outputs provide interpretable information for batch quality screening and the potential removal of identified stale berries during post-harvest processing.

## 2. Materials and Methods

### 2.1. Overview of the Designed Detection Method

**Figure 1:**
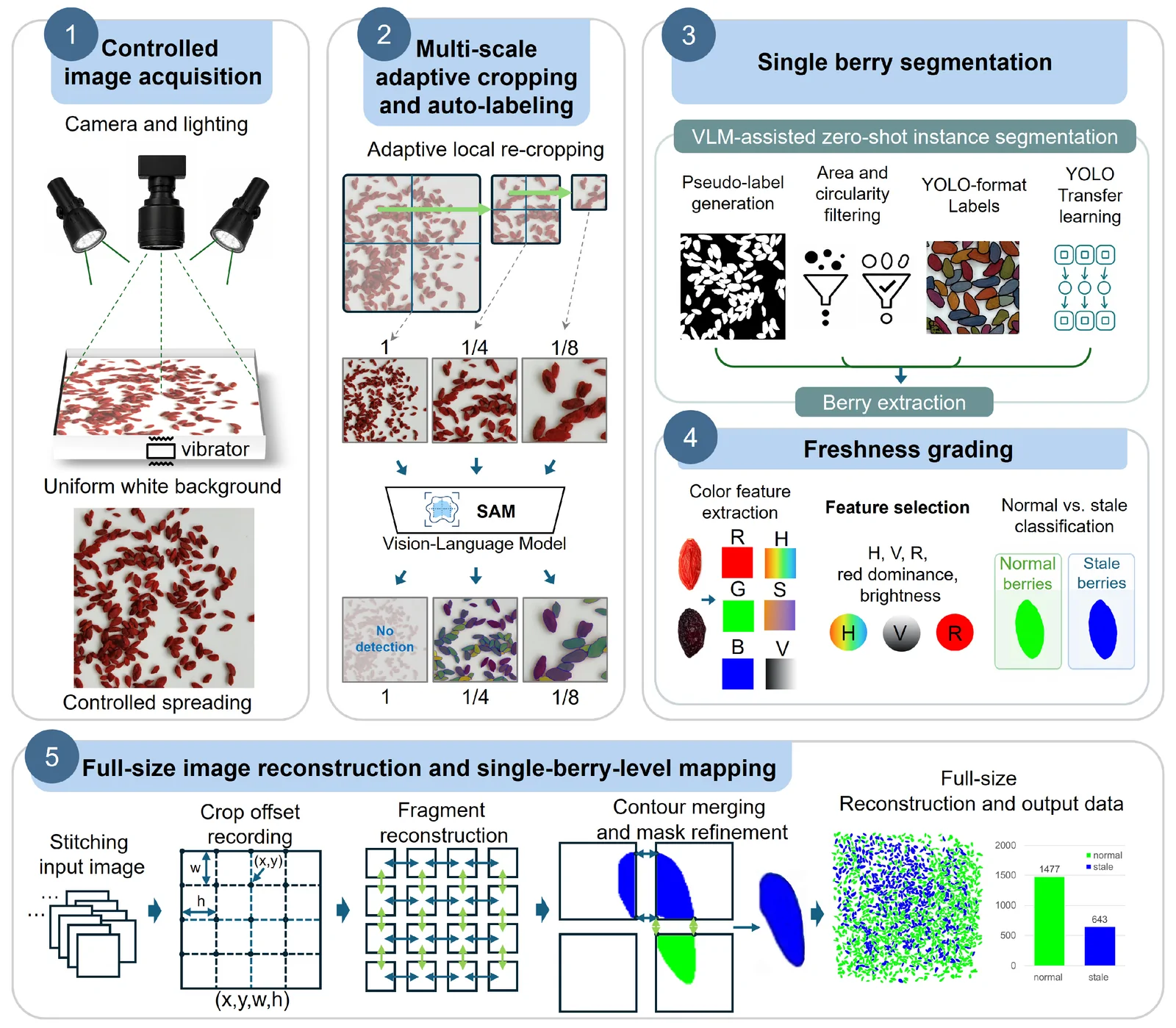
Procedure of the proposed method for detecting stale-berry mixing in dried goji berries, including standardized sample spreading and imaging, image acquisition, adaptive cropping, SAM annotation generation, YOLO-based berry localization, color-based freshness evaluation, reconstruction, and batch-level statistical reporting.

This study proposes an image-based quality assessment method for detecting stale-berry mixing in dried goji berries. As illustrated in Figure 1, the procedure starts from standardized single-layer spreading and fixed-condition imaging, followed by berry-region preparation, individual-berry localization, color-based freshness grading, reconstruction, and batch-level statistical reporting.

The process begins with a vibration-assisted sample spreading device, which is used to uniformly distribute goji berries into a single-layer configuration. Based on this preparation, images are acquired using a fixed top-view imaging system under controlled illumination and a uniform background, which guarantees consistency of the input data across all samples. The combination of SAM and an adaptive multi-scale cropping strategy is applied as a fully automatic annotation-generation tool. Lightweight YOLO segmentation models are trained using these pseudo labels for processing the high-density clustering of berries within each image using a zero-shot learning mode. Then, freshness grading was performed on YOLO-localized berry regions using a statistical color-feature grading rule. Finally, segmentation and grading results of cropped sub-images are combined to reconstruct the full-size berry freshness-grading map.

Overall, the method uses computer vision tools only to obtain reliable individual-berry regions. SAM provides annotations, YOLO provides berry locations, and the color-grading step provides freshness categories. The final outcome is an interpretable food-quality indicator, namely the stale-berry rate and its spatial distribution, rather than a single coupled vision model.

### 2.2. Data Collection Device and Samples

Images were acquired using a controlled imaging system for dried goji berries. An Apple iPhone 13 rear wide-angle camera with a 26 mm equivalent focal length and an aperture of f/1.6 was used. All images were captured in square mode at a resolution of 3024 × 3024 pixels and saved in JPEG format. The camera settings were fixed at ISO 50, exposure compensation of 0 EV, and a shutter speed of 1/100 s. White balance was kept constant during image acquisition. The smartphone was mounted vertically above the center of a 50 cm × 50 cm square sample container. The optical axis was approximately perpendicular to the sample plane. The distance between the camera lens and the sample plane was about 50–55 cm. A uniform matte-white background and constant LED lighting of about 5500 K were used during imaging. No explicit geometric calibration or color-chart-based calibration was performed in this study. Instead, fixed imaging geometry and constant illumination were used to keep the imaging conditions stable across samples.

The dried goji berry samples used in this study were obtained from commercial sources. Normal berries were provided by a dried goji berry supplier in Ningxia, China, and exhibited bright red color and good surface gloss. These berries were used as the normal class. Stale berries were obtained from commercially available dried goji berries that had been stored under room conditions for approximately two years after package opening. Compared with normal berries, stale berries showed visible quality deterioration, including darkened surface color and reduced surface gloss, and were used as the stale class. Since the samples used in this study were commercially obtained dried goji berries, detailed harvesting records were unavailable from the suppliers.

Before image acquisition, the berries were placed in a predefined 50 cm × 50 cm square container and spread over the full area with a vibration-assisted device. This process reduced local crowding and produced an approximately single-layer arrangement. The available sample pool included approximately 2 kg of normal berries and approximately 400 g of stale berries, from which experimental samples were manually selected. Under the maximum loading capacity of the imaging container, approximately 2500 berries could be accommodated in a single image. Except for one image containing the maximum number of berries, the number of berries used in each image was approximately 173 on average.

The computing platform was a desktop PC equipped with an Intel Core i7 processor, 32 GB RAM, and an NVIDIA RTX 4090 GPU. This system was used for image preprocessing, SAM pseudo-label generation, and YOLO model training and evaluation.

A total of 200 full-size images were collected. Direct application of SAM to the full-size images did not generate individual-berry masks due to dense berry distribution and severe touching between adjacent berries. Therefore, an adaptive multi-scale cropping strategy was applied before SAM inference to increase the relative berry size and improve local separability. The cropping process generated 600 sub-images, from which SAM successfully produced polygon masks. These masks were converted into pseudo-labels for YOLO model training (60%), validation (20%), and testing (20%).

An independent dataset of 30 full-size images was used for final evaluation. Each image was manually prepared as a mixed-berry sample with individual-level annotations and reserved exclusively for evaluating the complete workflow, including adaptive multi-scale cropping, coordinate remapping, duplicate suppression, fragment reconstruction, and batch-level statistical analysis. The stale-berry proportion in each sample was controlled at approximately 9–10% based on berry counts rather than sample mass. A predetermined number of stale berries was mixed with different numbers of normal berries across images, resulting in slight variations in the exact stale-berry proportion among individual samples. Across the 30 evaluation images, the pooled stale-berry proportion was 9.26%, calculated as the total number of stale berries divided by the total number of berries in the entire evaluation dataset. This controlled proportion was designed only for method validation and should not be interpreted as an estimate of stale-berry prevalence in commercial products. The evaluation dataset was excluded from detector training, color calibration, and parameter tuning.

Another image group (99 images) was used only for extraction of color statistics and determination of grading thresholds for the color-grading system. Among them, 83 images containing only normal goji berries were used to establish reference statistics for the normal class, while the remaining 16 images only stale berries were used to build statistics for the stale class. Individual berry regions were extracted for pixel-level analysis in RGB and HSV color spaces to extract color features, build statistical reference distributions, and determine standard parameters, feature weights, and classification thresholds.

### 2.3. Image Analysis Procedure for Individual-Berry Localization

To support berry-level quality assessment in dense dried-fruit images, the image-analysis procedure was designed to obtain separable and countable individual berry regions in the final evaluation images.

#### 2.3.1. Adaptive Multi-Scale Cropping and Pseudo-Label Generation

To generate instance-level pseudo-labels without prior task-specific manual annotation, SAM was introduced as an automatic mask generation model in this study [27]. It can generate instance masks from simple prompts without requiring prior task-specific manual annotation. However, when it was directly applied to full-size dense goji berry images, the output was unstable and many touching berries were merged. Therefore, adaptive multi-scale cropping was introduced before SAM inference. This step increased the relative berry size in each crop and improved local separation, which helped generate more reliable pseudo-labels for YOLO training.

SAM3 was expected to generate pseudo-labels for goji berry instance segmentation. However, for dense goji berry images, directly using SAM to generate pseudo-labels results in a substantial number of unrecognizable regions. Applying SAM to full-size images tends to produce pseudo-labels with severe local adhesion between adjacent berries, leading to insufficient separation and difficulty in individual instance segmentation.

To address this issue, a multi-scale adaptive cropping strategy is introduced. Very small cropped sub-images may increase the risk of missing targets, whereas very large cropped images may contain too many touching berries, making separation difficult. Therefore, a best image cropping scale is required. A set of representative sample images was manually selected. Empirical observations of berry density distribution and foreground proportion were used to guide the determination of image cropping rules.

The raw full-size image is defined as *I_0_*. In every image cropping, the image is divided into four equal parts. The cropped sub-images are fed into the SAM3 model for segmentation. If the number of segmented targets meets the designed rule, the current cropping scale is selected as the best. If not, these sub-images are cropped again, and the above steps are repeated until the requirements are met.

The geometric criteria, fragment reconstruction rules, and matching strategies used in this study were specifically designed for dense dried goji berry images based on spatial relationships between cropped regions and berry instances. Common mathematical operations (e.g., IoU calculation and overlap measurement) follow conventional definitions. The image cropping scale was determined based on two factors: $A_{f}$, the foreground pixels, and ${\bar{A}}_{n}$, the average pixel number of berries per image at the current scale (the image cropped *n* times). The condition for determining the rationality of automatic image segmentation results is expressed as

(1) $$T=\frac{A_{f}}{{\bar{A}}_{n}c}$$

where *c* is the detected instance by the SAM. If *T* > 1 and *T* < 10, the current cropping scale is the best. Otherwise, the image should be cropped again.

For a cropped sub-image $C_{k}$ with top-left offset $o_{k}=\left(x_{k},\;y_{k}\right)$, any local pixel or polygon vertex $p^{\prime }=\left(u,\;v\right)$ was mapped to the original image coordinate system as $p=p^{\prime }+o_{k}=(u+x_{k},v+y_{k})$. This mapping was used for output fusion, duplicate suppression, and final instance counting. Duplicate suppression was implemented in two steps. First, for two detections $B_{i}$ and $B_{j}$ from overlapping crops, the pair was treated as a duplicate when $IoU(B_{i},B_{j})>T_{IoU}$, and the lower-confidence record was removed. Second, for boundary-truncated berry records whose IoU did not exceed $T_{IoU}$ but whose boxes were adjacent to the same crop boundary, the pair was evaluated by the center distance $d_{c}$ and boundary-projection overlap $O_{ij}$; it was retained as one berry record only when $d_{c}<T_{c}$ and $O_{ij}>T_{o}$. Thus, IoU was the primary rule, whereas center distance and boundary overlap were auxiliary constraints for boundary ambiguity.

After adaptive multi-scale cropping, each sub-image was independently input into SAM to automatically form high-quality instance masks. This process improves pseudo-label robustness, reducing missed detections, and refines object boundaries. The final masks were converted into YOLO-compatible annotation format and used to supervise the training of an instance-segmentation model based on Ultralytics YOLO.

#### 2.3.2. YOLO Instance-Segmentation Model Training

YOLO segmentation models were trained to identify individual berries in quality-assessment images using the pseudo-labels generated by the SAM. The YOLO models were initialized using the pretrained weights yolov8n-seg.pt, yolo11n-seg.pt, yolo26n-seg.pt. The main training settings include epoch = 100 and resolution = 640. Other parameters for training were set to default values. Within the datasets for YOLO model construction, the training, validation, and test subsets were kept separate. The test subset was used only for performance evaluation and was not involved in model training, hyperparameter adjustment, or color-threshold calibration. For berry segmentation, widely used performance indicators including precision, recall, and mAP [19,24] were used to assess how well the system segmented and localized individual berries.

### 2.4. Freshness Grading Driven by Color Features

Normal and stale berries were differentiated according to literature-reported changes in surface appearance [28,29]. Compared with normal berries, stale berries generally exhibited increased dark-red, brown, or near-black areas, together with reduced apparent surface gloss. These differences were subsequently characterized using RGB- and HSV-derived color and brightness features [15,25]. Before color-feature extraction, all images were processed using a standardized preprocessing procedure to reduce noise and separate the berries from the background.

For freshness grading, binary classification accuracy was used to determine whether each detected berry was correctly classified as normal or stale. Before color-feature extraction, all images were processed using a standardized preprocessing procedure to reduce noise and separate the berries from the background. First, the images were resized to 800 × 800 pixels and smoothed using Gaussian filtering. The images were then converted from RGB to HSV color space. Pixels with V values greater than 200 were treated as high-brightness background and removed. The remaining pixels were screened using the predefined color ranges of normal and stale berries, and only pixels satisfying both the foreground and color criteria were retained. Finally, morphological closing and opening with a 5 × 5 structuring element were used to fill small holes and remove isolated noise, respectively [30]. The resulting clean berry regions were used for color-feature extraction and freshness classification.

During feature extraction, HSV and RGB pixel distributions were calculated for each berry region. The mean values of H, S, and V, the mean values of R, G, and B, the red-to-green ratio, the red-to-blue ratio, the normalized red–green contrast feature, and average brightness were then derived. The color indices were calculated based on commonly used RGB statistical descriptors [15,25,30]. The normalized red–green contrast feature was defined as

(2) $$C_{RG}=\frac{R-G}{R+G+\varepsilon }$$

where ε is a small constant introduced to avoid division by zero.

The above color-related parameters were subjected to statistical correlation analysis on the 99-image calibration dataset, and variables showing strong association with quality grading were selected as the primary indicators for subsequent freshness classification. After calibration, these selected indicators and their weights were kept unchanged for the 30-image final evaluation dataset.

At the individual-berry freshness-grading stage, a color similarity score was calculated for each YOLO-detected berry region in the 30-image final evaluation dataset using the fixed reference means of normal and stale groups obtained from the 99-image calibration dataset. H, V, R, normalized red–green contrast, and brightness were selected as core variables and assigned feature weights to calculate similarity scores for reference-category assignment. The resulting color-grading step is a statistical feature-based rule rather than an end-to-end learned model. A berry was classified as normal when its similarity to the normal reference exceeded that of the stale reference and surpassed a predefined threshold; otherwise, it was classified as stale.

Freshness grading was first performed on sub-images generated from the 30 final evaluation images. The crop offset was recorded and used to map berry fragments back to the original image coordinate system after detection and grading. Reconstruction was then performed before whole-image visualization to reduce boundary artifacts and coordinate inconsistencies caused by overlapping crops.

### 2.5. Freshness-Grading Distribution Map Reconstruction

Because berries located on crop boundaries could be divided into multiple fragments, a fragment reconstruction procedure was introduced before berry counting and visualization. Fragments were first filtered according to their relative area [31,32]. $A_{i}$ denotes the area of fragment i, and $A_{avg}$ denotes the average area of a single berry. A fragment filtering criterion was designed in this study based on the relative area of each fragment:

(3) $$F_{i}=\left\{\begin{array}{ll} discard, & A_{i}<T_{\min}\\ retain, & A_{i}>T_{\max}\\ candidate, & T_{\min}\leq A_{i}\leq T_{\max} \end{array}\right.$$

where $T_{\min}$ and $T_{\max}$ are the lower and upper area thresholds, respectively. The threshold values were determined using the calibration dataset and remained unchanged during final evaluation. Very small fragments were removed because they contained limited appearance information and contributed little to reconstruction. Large fragments were considered sufficiently complete and were retained directly. The remaining fragments were treated as candidate fragments for reconstruction.

For each candidate fragment, boundary line segments caused by cropping were identified and recorded in the original image coordinate system. Fragments separated by vertical crop boundaries were checked first, followed by fragments separated by horizontal boundaries, consistent with the spatial adjacency of the crop regions.

Fragment matching was performed only between adjacent crop regions that shared the same crop boundary. The primary matching criterion was the overlap of boundary-segment projections. To identify whether fragments from adjacent crop regions belonged to the same berry, an overlap-based matching criterion was developed in this study. The overlap ratio was defined as

(4) $$O_{seg}=\frac{L_{overlap}}{\min(L_{1},L_{2})}$$

where $L_{overlap}$ is the overlapping length between two projected line segments and $L_{1},L_{2}$ are the lengths of the corresponding line segments. Two fragments were considered matched when

(5) $$O_{seg}\geq T_{overlap}$$

where $T_{overlap}$ is the overlap threshold.

Fragments were assigned to the same reconstructed-berry group $G_{k}$ only when they came from adjacent crop regions sharing the same crop boundary and satisfied the matching conditions: boundary-projection overlap $O_{ij}>T_{o}$, center distance $d_{c}<T_{c}$, and acceptable area consistency. Fragments that did not satisfy these conditions were retained as independent-berry instances. This membership rule was applied before label fusion and allowed crop-split berries, including berries crossing vertical or horizontal boundaries, to be counted as one berry.

After fragment grouping, contours from fragments within the same group were merged to form one berry mask. Matched boundary endpoints were connected with the non-boundary contours. This step corrected crop-boundary fragmentation and reduced boundary artifacts in the final masks.

After the reconstructed-berry groups $G_{k}$ had been defined, conservative label fusion was applied only within each group. Let $Label_{i}$ denote the freshness label of fragment. A conservative label fusion rule was developed in this study to determine the final label of each reconstructed berry:

(6) $$Label_{\mathrm{berry}}=\left\{\begin{array}{ll} stale, & \exists i,Label_{i}=stale\\ normal, & otherwise \end{array}\right.$$

For each reconstructed-berry group $G_{k}$, if any fragment in the group was classified as stale, the reconstructed berry was labeled as stale; otherwise, it was labeled as normal. This one-vote veto rule was applied after fragment membership was determined to reduce the risk of missing stale berries during quality inspection.

Finally, freshness labels were mapped onto the reconstructed-berry masks to generate complete grading maps. The grading result at pixel position (*x*, *y*) was expressed as

(7) $$M(x,y)=Label_{berry},(x,y)\in Mask_{berry}$$

where $M_{rec}(x,y)$ denotes the reconstructed-berry mask at pixel position (*x*, *y*). The resulting annotated images show the spatial distribution of normal and stale berries and support berry counting and batch-level mixing-risk assessment.

## 3. Results

### 3.1. Output of Adaptive Multi-Scale Cropping and SAM-Assisted Segmentation

When SAM was directly applied to full-size goji berry images, the segmentation results were unsatisfactory in dense scenes. Severe adhesion between adjacent berries frequently occurred, leading to merged instances, repeated boundary detections, and local missed targets. These errors became more pronounced when inter-berry spacing was small and local occlusion was strong, indicating that direct full-image processing was insufficient for stable instance localization in dense berry scenes. To address this limitation, an adaptive multi-scale cropping strategy was introduced before SAM inference.

**Figure 2:**
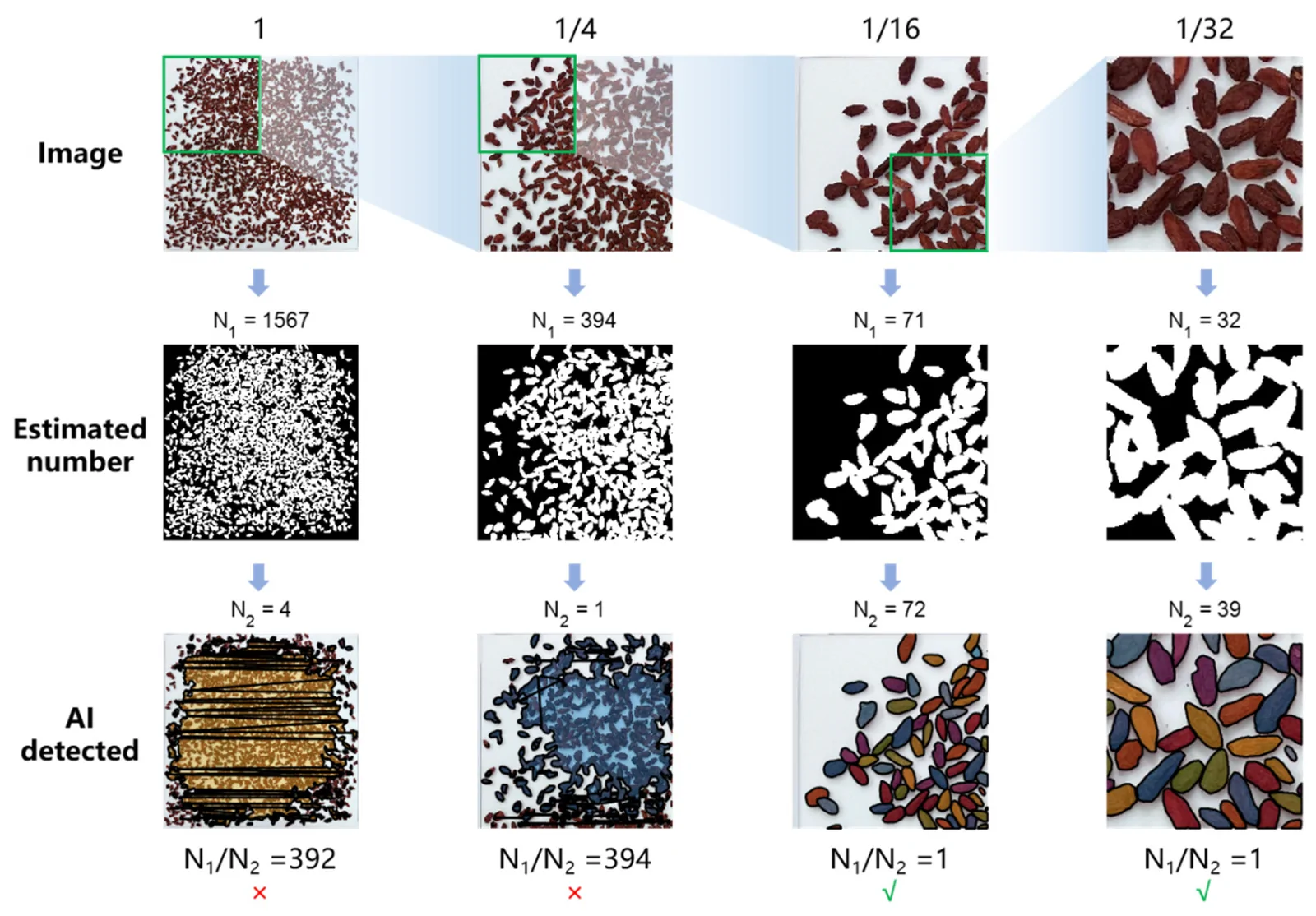
Effect of different cropping scales on dense-scene goji berry segmentation. From left to right, the columns represent progressively reduced cropping scales, and each green box marks the display range of the next smaller cropping scale within the current scale. The first row shows the original image or selected crop regions, the second row presents the corresponding binary foreground masks, and the third row shows the SAM segmentation results overlaid on the images. Red crosses and green check marks indicate inadequate and acceptable instance separation, respectively.

To investigate the influence of crop scale on segmentation performance, representative dense-scene samples were analyzed under different cropping scales (1, 1/4, 1/16, and 1/64), as shown in Figure 2. As the number of times the image was cropped increased, the proportion of pixels of a single berry in the entire image increased. The relative separation between adjacent berries became clearer. At the original scale and the 1/4 scale, severe overlap and under-localization were still observed. A group of berries was detected as one sample, but the individuals in this group were not separate.

$N_{1}$ denotes the manual berry count within the cropped image, and $N_{2}$ denotes the detected berry count by the SAM. The ratio $N_{1}/N_{2}$ was used to describe counting completeness. Values closer to 1 indicate better local separation and counting consistency. The ratio $N_{1}/N_{2}$ for 1 to 10 was acceptable in this study.

From the images, it is evident that at large scales (1 and 1/4), dense berry regions exhibit severe overlap and incomplete separation, resulting in significant discrepancies between the actual and detected berry counts ($N_{1}/N_{2}>1$). At a scale of 1/16, berries are clearly separated, overlaps are minimized, and $N_{1}/N_{2}$ approaches 1, indicating accurate segmentation. Further reducing the cropping scale to 1/64 does not substantially improve separation, while increasing the number of sub-images and computational cost. These observations indicate that an appropriate crop scale is important for balancing localization quality and computational efficiency in dense scenes. The adaptive multi-scale cropping strategy is therefore crucial for balancing accurate berry separation and computational efficiency.

Based on this analysis, the adaptive multi-scale cropping strategy was applied to the 200 original images because direct SAM inference on full-size images failed to provide reliable berry-level masks. The strategy generated 29,766 candidate masks, which were further filtered and converted into pseudo-labels for YOLO training.

After geometric filtering, the masks generated from the adaptively cropped sub-images were retained as pseudo-labels for YOLO training. To assess annotation reliability, a randomly selected 5% subset of the generated masks was manually inspected. The inspection showed that, compared with direct SAM inference on full-size images, SAM applied to multi-scale cropped sub-images achieved higher localization accuracy and better instance separation in dense scenes, producing masks that were more consistent with the actual berry boundaries. Overall, adaptive multi-scale cropping enabled SAM to generate sufficiently accurate and reliable annotations, thereby providing a high-quality training set for subsequent YOLO training and lightweight detection.

### 3.2. YOLO Models Fine-Tuned Using Pseudo Labels

Using pseudo-labels generated by the SAM, the YOLO segmentation model achieved stable detection performance in processing images with densely arranged goji berries. During the whole image processing and segmentation stage, no manual annotation was required, making the proposed method a zero-shot deep learning approach.

As listed in Table 1, different versions of YOLO models were tested and compared. All these models achieved good segmentation performance (mAP_50_ > 0.94). The YOLO11n-seg realized the best instance-segmentation performance, with precision = 0.953, recall = 0.951, mAP_50_ = 0.960, and mAP_50-95_ = 0.846.

**Figure 3:**
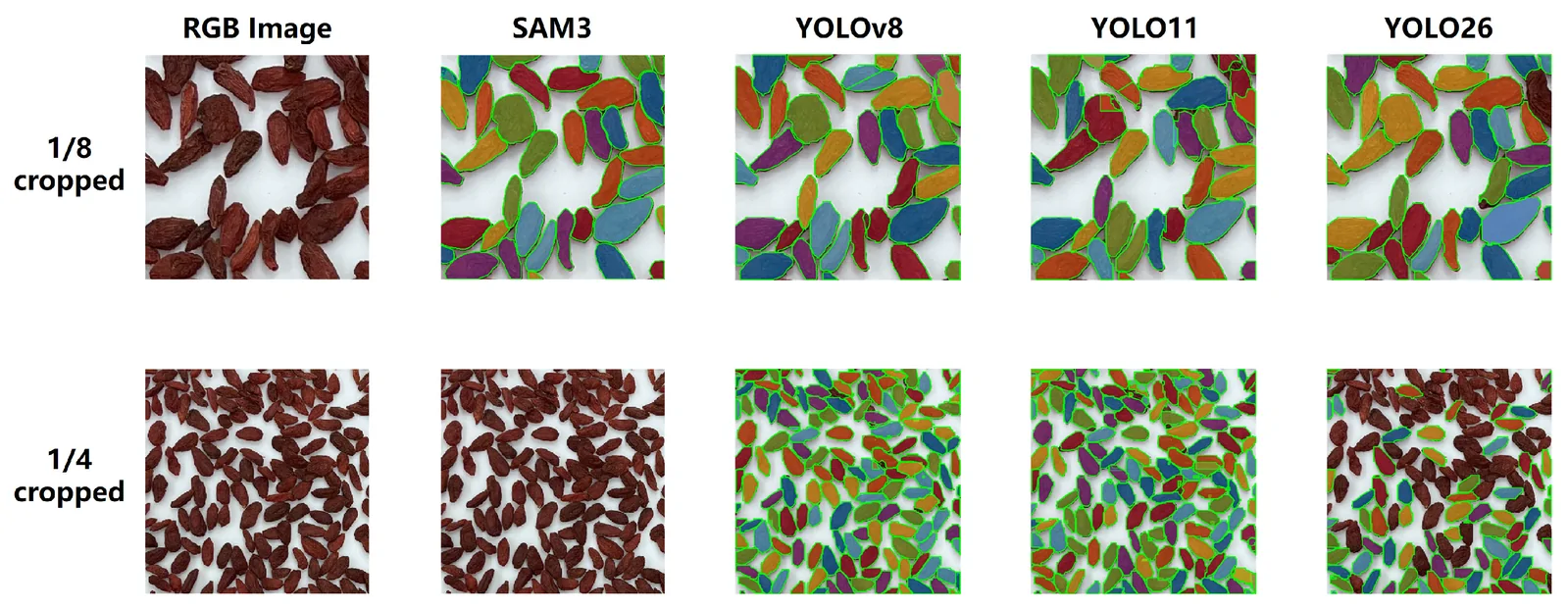
Visual comparison of goji berry instance-segmentation results obtained using SAM3 and different YOLO segmentation models at two cropping scales. Colored masks denote individual berry instances, and green contours indicate the predicted instance boundaries.

When processing images with touching goji berries, the trained YOLO segmentation model could still produce stable multi-instance-segmentation results. As illustrated in Figure 3, most berry instances were successfully extracted with a clear boundary line. For processing cropped sub-images of 1/8 scale, the SAM3 generated perfect masks with smooth boundaries. YOLO-series models also showed satisfactory results, but some duplicate detections and incomplete detections were observed. When processing the cropped sub-images of 1/4 scale, the SAM3 could not find any berry in the RGB image. However, YOLO-series models achieved relatively good segmentation performance. The SAM3 was a trained generic vision model, which could not extract berry instances at this scale. These YOLO models could learn berry-related information using pseudo-labels at other cropping scales, and achieve better performance for multi-scale berry image processing. More specifically, for example, the YOLO models could learn the features of berries at 1/4 scale using annotated images of 1/8 scale with the help of data augmentation (random resizing).

Adaptive multi-scale preprocessing further improved segmentation stability in dense scenes. The mask outputs from individual crops were mapped back to the original image coordinate system, and neighboring crop results were merged before final counting. This step reduced duplicate berry masks caused by crop-boundary splitting and ensured that each berry was represented by one final segmented region in the reconstructed visualization.

Overall, the combination of SAM-generated annotations and YOLO-based instance segmentation can provide stable individual-berry separation in dense scenes while substantially reducing the need for manual labeling.

### 3.3. Freshness Grading of Individual Berries and Batch-Level Estimation of Mixing Ratio

**Figure 4:**
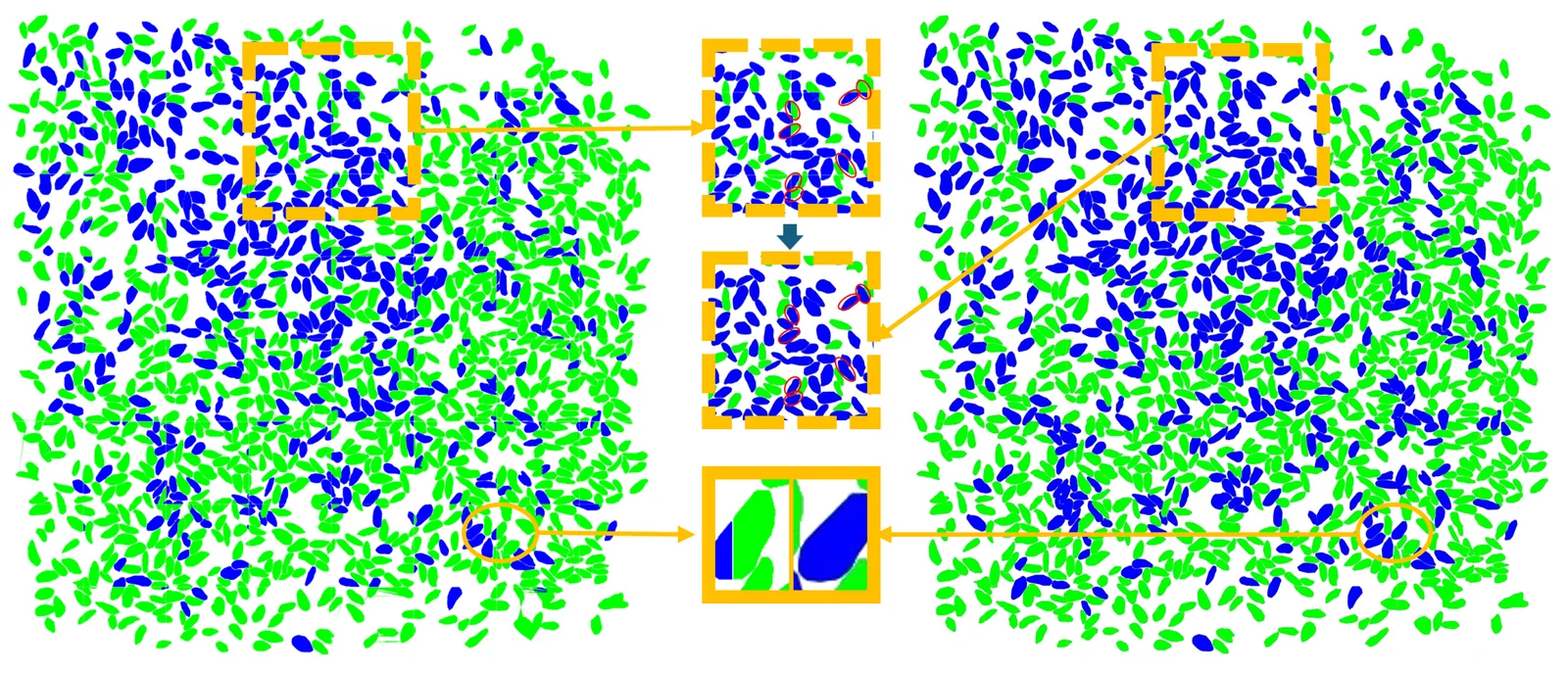
Comparison of the directly stitched freshness-grading map and the reconstructed system output. Reconstruction corrected inconsistent colors among berry fragments and removed white seams along crop boundaries. Green and blue regions indicate normal and stale berries, respectively. The red circles at the same position correspond to the identical wolfberry fruit in the two panels, which serves as a visual comparison of the wolfberry before and after reconstruction processing.

After YOLO localization, berry freshness was assigned by the color statistical rule described in Section 2.4. In the reconstructed outputs used for batch-level analysis, 4850 berries were counted in total, including 4401 normal berries and 449 stale berries. The mean stale-berry ratio was 9.26%. These results show that berry-level grading can be translated into clear batch-level freshness indicators. Representative freshness-grading results are shown in Figure 4. Normal and stale berries were separated clearly under dense-scene conditions, and the spatial distribution of each class remained stable after reconstruction. These results show that the color statistical rule can provide interpretable berry-level freshness outputs for mixed goji berry samples.

**Figure 5:**
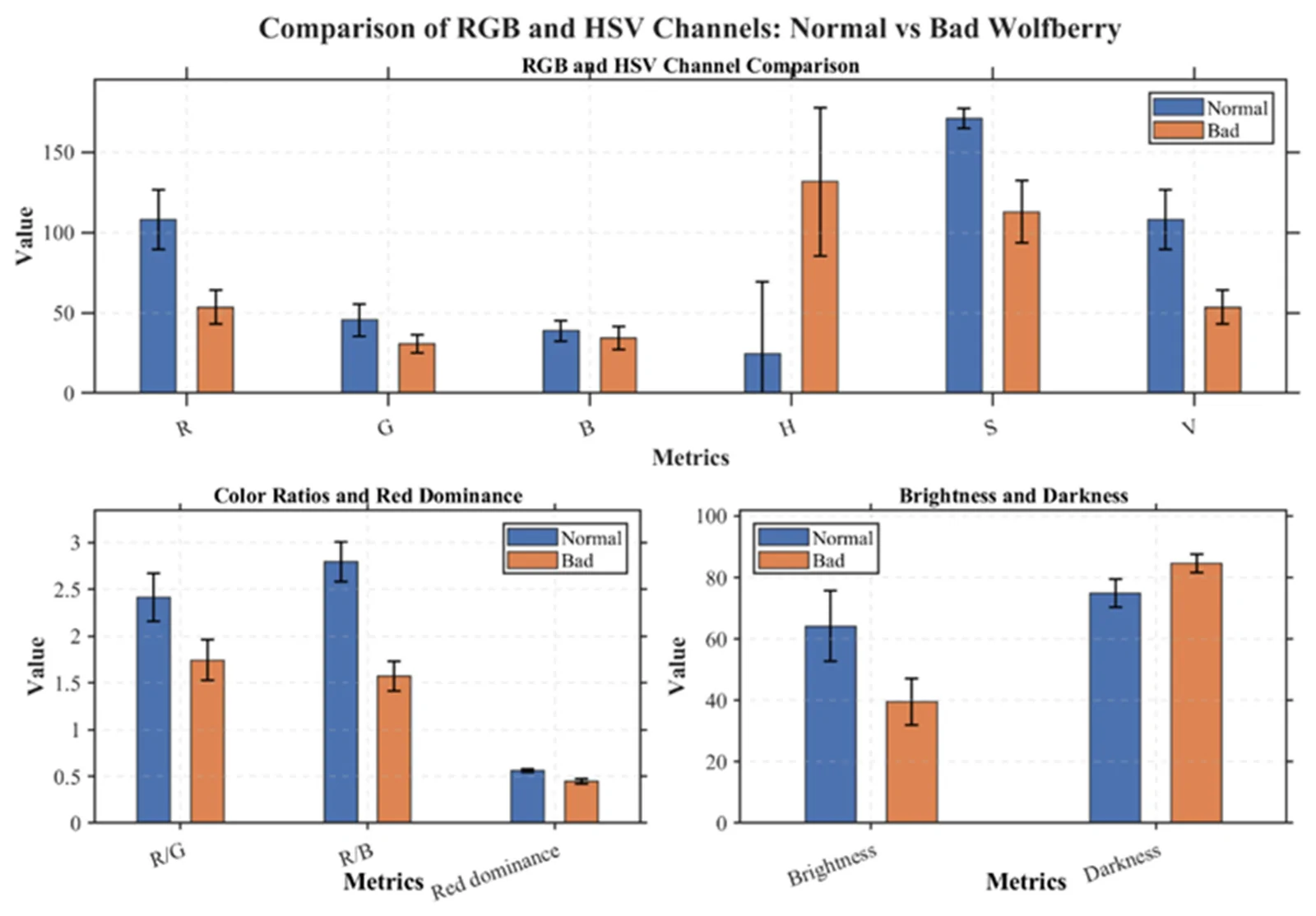
Comparison of RGB- and HSV-derived color features between normal and stale dried goji berries. The upper panel shows the R, G, B, H, S, and V channel values; the lower-left panel presents the R/G and R/B ratios and red-dominance index; and the lower-right panel shows the brightness and darkness features.

To further evaluate the discriminative capability of color statistical features, RGB and HSV analyses were conducted for both normal and stale-berry groups. As shown in Figure 5 and Table 2, normal berries had higher saturation, higher brightness, higher V values, and stronger red dominance than stale berries. At the image level, mean V decreased from 112.78 to 62.55, and mean brightness decreased from 67.16 to 42.22. These trends are consistent with previous color-based fruit quality studies [15,25,28]. H, V, R, normalized red–green contrast, and brightness were retained as the final variables for the statistical grading rule, with weights of 0.5, 3.5, 3.5, 2.0, and 2.0, respectively. The decision threshold for the normal class was set to 2.5.

To improve counting consistency in dense scenes, reconstruction was applied after adaptive multi-scale cropping. Berries that crossed crop boundaries could be split into adjacent fragments, which introduced boundary artifacts and repeated detections after coordinate fusion. Crop-level localization and grading results were therefore mapped back to the original image coordinate system and merged according to their recorded crop positions.

**Figure 6:**
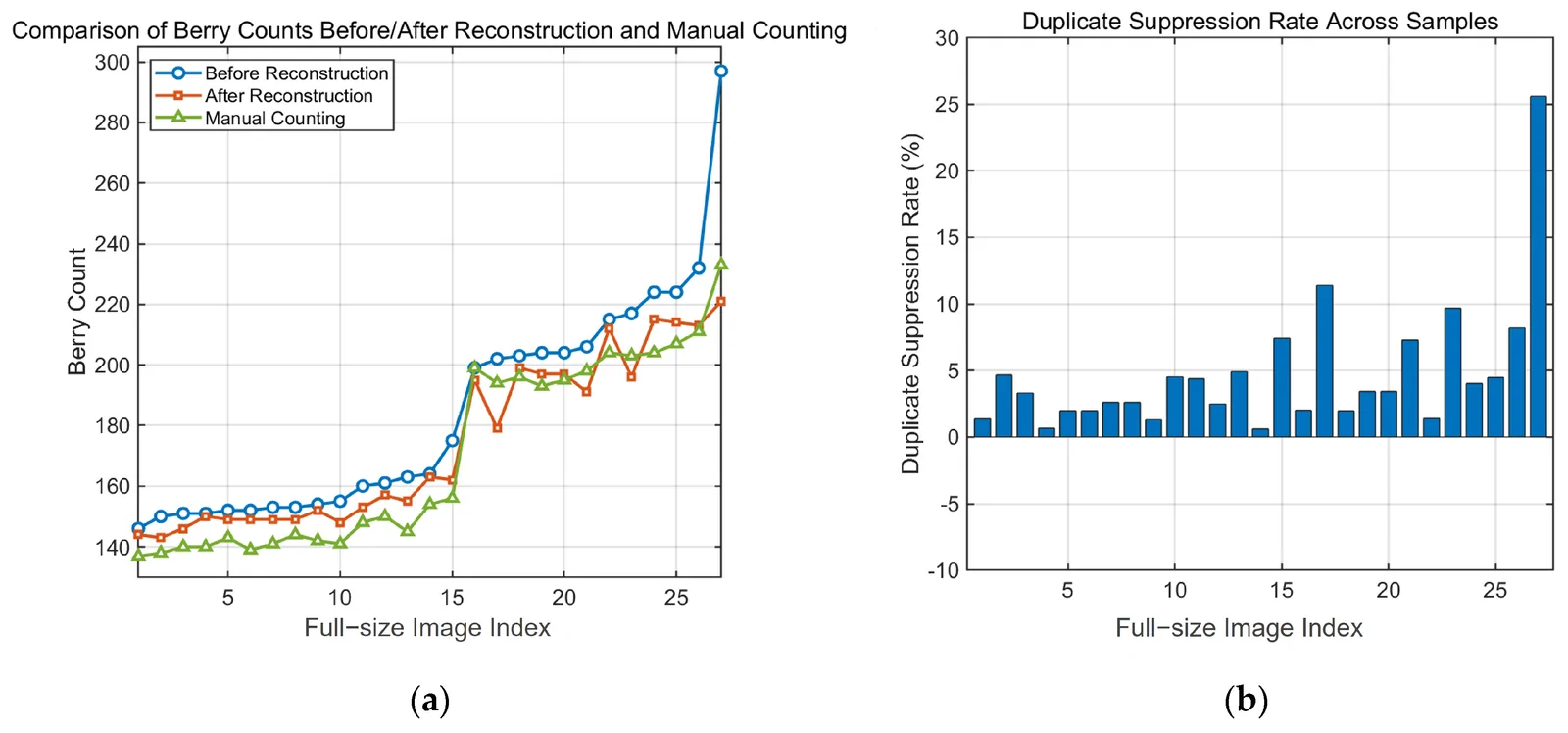
Reconstruction and duplicate-suppression results following adaptive multi-scale cropping. (**a**) Comparison of berry counts before and after reconstruction, with manual counts included as the reference. (**b**) Duplicate-suppression rates across the evaluated full-size images. The reconstruction procedure reduced duplicate detections arising from crop-boundary splitting.

As shown in Figure 6a, reconstruction improved counting consistency by merging fragments that belonged to the same berry. For example, in a representative case using a 3072 × 3072 image reconstructed from four 1536 × 1536 crops, 173 mapped fragments were first projected back to the original coordinate system. Among them, 18 cross-boundary pairs were successfully matched, resulting in 144 final berries. This case reflects the overall trend, where the mean berry count decreased from 182.46 before reconstruction to 173.21 after reconstruction.

The stale-berry ratio remained stable after reconstruction. The mean stale-berry ratio changed only from 9.90% before reconstruction to 9.26% after reconstruction. In a representative manual count check of final outputs, the mean absolute relative counting error was 1.61%, which indicates good agreement with visual inspection.

The suppression rates in Figure 6b further show the value of the reconstruction step. Most samples showed positive duplicate suppression, and several crowded samples exceeded 20%. The mean duplicate-suppression rate was 4.31%. In the representative reconstruction case, matched fragment pairs showed a mean boundary-overlap ratio of 0.943 and a mean center distance of 4.00 px, which supports the stability of the matching rule.

Overall, the experimental results show that the proposed method can provide stable berry-level freshness grading and stable batch-level estimation of stale-berry proportion under mixed-sample conditions.

## 4. Discussion

### 4.1. Advantages of the Designed Method

Compared with conventional batch-level inspection methods and previous vision-based approaches, the proposed framework is better suited for hidden stale-berry detection in mixed dried goji berry samples. Traditional fruit and dried-product quality evaluation methods mainly rely on physicochemical analysis, spectroscopy, or whole-image grading, which are often unable to identify hidden abnormal berries within visually acceptable batches [5,6,7,8]. However, these methods generally provide batch-level information or image-level classification results and cannot identify the location and proportion of individual abnormal berries within mixed samples. Similarly, recent deep learning-based fruit quality studies still mainly focus on image-level classification or maturity grading rather than fine-grained particle-level abnormality analysis [12,14,15,16]. In contrast, the proposed framework transforms traditional group-level grading into berry-level freshness evaluation, which is more suitable for detecting hidden stale-berry adulteration during the final packaging stage.

A major advantage of the workflow is that it separates annotation generation, localization, and freshness grading into clear steps. Dense goji berry images often contain severe overlap, adhesion, and local occlusion, which makes manual pixel-level annotation difficult and time-consuming. In this study, SAM was used only as an annotation-generation tool, and the retained annotations were used as pseudo-labels for YOLO training. YOLO then provided berry localization during sample evaluation. This design reduced manual annotation cost while maintaining stable dense-scene detection performance, with a precision of 0.953 and an mAP_50_ of 0.960.

Another important advantage of the proposed framework is its robustness in dense small-object scenes. Dense agricultural targets frequently suffer from overlap, touching boundaries, and local occlusion, which can lead to merged masks and missed detections during segmentation and localization [18,23,33]. Previous studies have shown that dense small-object detection remains a major challenge even for advanced vision systems because boundary adhesion and object aggregation significantly reduce segmentation stability [34]. In the present study, adaptive multi-scale cropping enlarged the relative berry size within local regions and reduced inter-object overlap, thereby improving boundary separability in dense scenes. For some images of relatively large scale, the berries become ‘Small’ and could not be segmented by the SAM. But the fine-tuned YOLO models, which were trained using several fixed scale image–label pairs and random resizing augmentation, could adapt to image processing at other scales [24]. This presented method simultaneously retains SAM’s zero-shot segmentation characteristic and YOLO’s multi-scale adaptation advantages.

Reconstruction and spatial merging further suppressed duplicate detections caused by crop-boundary splitting. Experimental results showed that the average berry count decreased from 182.46 before reconstruction to 173.21 after reconstruction, corresponding to an average duplicate-suppression rate of 4.31%. These results indicate that the proposed adaptive restructuring strategy effectively improved dense-scene counting consistency while maintaining stable localization performance.

The proposed framework also provides a more interpretable freshness-grading strategy than conventional end-to-end black-box classification models. Many recent deep learning-based freshness-detection methods mainly rely on implicit feature learning and therefore provide limited physical interpretability for practical quality supervision [14,15,16]. In contrast, the present study used RGB-HSV statistical features for berry-level freshness grading. Normal berries generally showed higher brightness and stronger red-related responses, whereas stale berries showed lower V values and darker appearances. At the image level, mean V decreased from 112.78 in normal berries to 62.55 in stale berries, and mean brightness decreased from 67.16 to 42.22. These changes are consistent with human visual perception of freshness degradation [15,28] and therefore provide interpretable grading evidence for quality supervision.

Finally, the workflow supports batch-level stale-berry proportion estimation in addition to berry-level freshness grading. Existing fruit quality studies mainly focus on defect classification or maturity recognition [11,12,14,15,16], whereas quantitative estimation of abnormal-particle proportions within mixed batches is reported less often. In practical quality inspection, however, the proportion of suspicious particles is often more meaningful than simple binary batch classification. In the reconstructed outputs used for final batch-level analysis, 4850 berries were counted, including 449 stale berries, and the mean stale-berry ratio was 9.26%. These results show that the workflow can provide stable quantitative freshness evaluation under controlled imaging conditions.

To summarize the role of each step in the workflow, Table 3 reports the main function and the directly relevant quantitative output for the annotation, localization, and grading stages.

### 4.2. Practical Applications

The detected berry locations, freshness classification results, and reconstructed quality maps provide more than simple quality evaluation outputs. At the batch level, the estimated stale-berry proportion can be used as an objective indicator for quality grading and acceptance decisions during packaging or inspection. At the individual-berry level, the spatial information of detected stale berries provides potential guidance for targeted removal using automated sorting devices, such as vacuum suction or robotic picking systems. Therefore, the proposed framework may serve as an information source for intelligent quality control, including batch screening, selective removal, and traceability management of dried-fruit products.

In practical quality control scenarios, the estimated stale-berry proportion can be used as a quantitative indicator for preliminary batch classification and quality evaluation [10,11,12]. For example, batches with different levels of stale-berry occurrence can be assigned different quality grades or subjected to different handling strategies. Meanwhile, the spatial localization information generated by the proposed framework may provide guidance for targeted removal of identified stale berries using automated sorting devices, such as robotic manipulators or suction-based separation systems.

Therefore, the proposed framework is not intended to replace standardized quality evaluation methods but rather to serve as a non-destructive visual screening tool that provides objective information for quality inspection, traceability, and further automated processing [13].

### 4.3. Limitations and Future Works

Several limitations should be noted. First, the proposed workflow was validated under controlled two-dimensional imaging conditions. Although this design ensured stable image acquisition, it does not fully represent complex industrial environments with stronger stacking, uneven layer thickness, or severe occlusion. Future studies should investigate multi-view imaging or depth-assisted reconstruction strategies to improve the representation and localization of overlapping berries.

Second, the current dataset was collected under controlled experimental conditions and mainly included predefined normal and stale-berry samples. Future studies should establish larger datasets covering more diverse product sources, storage conditions, and quality variations to further evaluate the robustness of the proposed framework in practical quality control scenarios.

Third, the current freshness-grading strategy still relies on manually selected color features, empirical weights, and a fixed decision threshold. Although this design improves interpretability, it may limit adaptability under variations in illumination, product sources, and quality characteristics. Future work could explore data-driven feature selection and adaptive decision strategies.

In addition, the current study mainly evaluated stale-berry proportion using berry counts under controlled imaging conditions. Future work should establish the relationship between berry-level results and mass-based quality standards and validate the workflow under more variable industrial conditions. Furthermore, integrating complementary quality information, such as spectral characteristics or physicochemical indicators, may help develop a more comprehensive freshness assessment framework.

## 5. Conclusions

In this study, a vision-based workflow was developed for detecting hidden stale-berry adulteration in densely arranged dried goji berries. By integrating adaptive multi-scale cropping, SAM-assisted pseudo-label generation, berry-level localization, and RGB–HSV statistical grading, the proposed workflow enabled individual-berry detection, freshness classification, and stale-berry proportion estimation. The adaptive cropping strategy improved berry separation in dense scenes, while the reconstruction procedure corrected fragmented predictions and generated complete quality assessment maps. The proposed method achieved reliable detection and grading performance, with an mAP_50_ of 0.960 and a mean absolute counting error of 1.61%. More importantly, the berry-level classification results were converted into quantitative stale-berry proportions, providing an interpretable indicator for evaluating the quality status of dried goji berry samples. Therefore, the proposed workflow offers a non-destructive and interpretable approach for quality screening before packaging and sale. Future studies should validate the framework under more complex industrial conditions and explore additional visual or spectral information to improve robustness.

## Figures and Tables

**Table 1:** Segmentation performances of different YOLO models.

Model	Precision	Recall	mAP_50_	mAP_50-95_
YOLOv8n-seg	0.953	0.951	0.960	0.846
YOLO11n-seg	0.949	0.943	0.957	0.841
YOLO26n-seg	0.928	0.916	0.945	0.801

**Table 2:** Exact image-level RGB, HSV, and derived color features for normal and stale berries.

Metric	Normal (*n* = 83)	Stale (*n* = 16)	Metric	Normal (*n* = 83)	Stale (*n* = 16)
R	112.78 ± 10.56	62.55 ± 7.32	R/G	2.35 ± 0.20	2.13 ± 0.44
G	48.46 ± 6.32	30.12 ± 4.95	R/B	2.81 ± 0.16	1.92 ± 0.49
B	40.42 ± 4.43	30.12 ± 4.95	Red dominance	0.56 ± 0.02	0.50 ± 0.05
H	8.99 ± 21.63	147.25 ± 29.12	Brightness	67.16 ± 6.96	42.22 ± 4.70
S	170.75 ± 4.94	137.22 ± 24.74	Darkness (%)	73.66 ± 2.74	83.46 ± 1.83
V	112.78 ± 10.56	62.55 ± 7.32			

**Notes.** Values are presented as mean ± SD. All statistics were calculated at the image level. R, G, and B represent the red, green, and blue channel values, respectively. H, S, and V represent hue, saturation, and value, respectively. R/G and R/B are channel ratios. Red dominance, brightness, and darkness are derived indices from the image analysis pipeline. Darkness is expressed as a percentage to match the figure.

**Table 3:** Role summary of the main steps in the proposed workflow.

Step	Main Output	Key Result	Metric/Rule	Role in Workflow
SAM annotation	Pseudo-labels (masks)	29,766 candidate masks; 148.8 masks per image	5% manual check	Berry annotation
YOLO segmentation	Instance-level masks	Precision 0.953; recall 0.951; mAP_50_ 0.960; mAP_50-95_ 0.846	Segmentation metrics	Berry localization
Color grading	Normal/stale labels and batch summary	4850 berries; 449 stale berries; mean stale ratio 9.26%	Statistical color rule	Berry classification

**Notes.** The table summarizes the role of each step in the workflow rather than treating them as one coupled model. SAM was used only for annotation generation and therefore is summarized by annotation quantity and manual-check consistency. YOLO was used only for berry localization. The color-grading step was used only for freshness classification and batch-level output interpretation.

## Data Availability

The original contributions presented in the study are included in the article; further inquiries can be directed to the corresponding authors.

## References

[1] Tian Y., Xia T., Qiang X., Zhao Y., Li S., Wang Y., Zheng Y., Yu J., Wang J., Wang M. (2022). Nutrition, Bioactive Components, and Hepatoprotective Activity of Fruit Vinegar Produced from Ningxia Wolfberry. Molecules.

[2] Shi X., Wang X., Zheng Y., Fu L. (2024). Advances in the Study of Bioactive Compounds and Nutraceutical Properties of Goji Berry (*Lycium barbarum* L.). Appl. Sci..

[3] Li T., Luo Y., Zhao T. (2024). Research on enhancing the efficiency of food safety sampling inspections in China based on Pareto’s law. J. Sci. Food Agric..

[4] Zhang Y., Ma L., Yi W., Wu L. (2024). Research on the adulteration of *Lycium barbarum* based on hyperspectral imaging technology combined with deep learning algorithm. J. Food Compos. Anal..

[5] Nespeca M.G., Vieira A.L., Júnior D.S., Neto J.A.G., Ferreira E.C. (2020). Detection and quantification of adulterants in honey by LIBS. Food Chem..

[6] Laborde A., Puig-Castellví F., Jouan-Rimbaud Bouveresse D., Eveleigh L., Cordella C., Jaillais B. (2021). Detection of chocolate powder adulteration with peanut using near-infrared hyperspectral imaging and Multivariate Curve Resolution. Food Control.

[7] Patel D., Bhise S., Kapdi S.S., Bhatt T. (2024). Non-destructive hyperspectral imaging technology to assess the quality and safety of food: A review. Food Prod. Process. Nutr..

[8] Chen X., Zhao X., Jiao L., Xing Z., Dong D. (2024). Infrared microspectroscopy and machine learning: A novel approach to determine the origin and variety of individual rice grains. Agric. Commun..

[9] Liu Y., Zhou S., Wan Z., Qiu Z., Zhao L., Pang K., Li C., Yin Z. (2023). A Self-Supervised Anomaly Detector of Fruits Based on Hyperspectral Imaging. Foods.

[10] Hassoun A., Jagtap S., Garcia-Garcia G., Trollman H., Pateiro M., Lorenzo J.M., Trif M., Rusu A.V., Aadil R.M., Šimat V. (2023). Food quality 4.0: From traditional approaches to digitalized automated analysis. J. Food Eng..

[11] Xiao Z., Wang J., Han L., Guo S., Cui Q. (2022). Application of Machine Vision System in Food Detection. Front. Nutr..

[12] Shen C., Wang R., Nawazish H., Wang B., Cai K., Xu B. (2024). Machine vision combined with deep learning–based approaches for food authentication: An integrative review and new insights. Compr. Rev. Food Sci. Food Saf..

[13] Wu L., Zhu L., Weng H., Chen G., Liu H., Liu Y., Ye D. (2026). Edge computing-based computer vision and deep transfer learning for high-throughput assessment of *Aspergillus flavus* infection in crop seeds. Plant Phenomics.

[14] Azimi N., Rezaei D.M. (2024). Automated Defect Detection and Grading of Piarom Dates Using Deep Learning (Version 1). arXiv.

[15] Sarkar T., Mukherjee A., Chatterjee K. (2021). Supervised Learning Aided Multiple Feature Analysis for Freshness Class Detection of Indian Gooseberry (*Phyllanthus emblica*). J. Inst. Eng. (India) Ser. A.

[16] Rizzo M., Marcuzzo M., Zangari A., Gasparetto A., Albarelli A. (2022). Fruit Ripeness Classification: A Survey (Version 3). arXiv.

[17] Ma J., He Y., Li F., Han L., You C., Wang B. (2024). Segment anything in medical images. Nat. Commun..

[18] Amir S.B., Horio K. (2024). YOLOv8s-NE: Enhancing Object Detection of Small Objects in Nursery Environments Based on Improved YOLOv8. Electronics.

[19] Wang C.-Y., Bochkovskiy A., Liao H.-Y.M. (2022). YOLOv7: Trainable bag-of-freebies sets new state-of-the-art for real-time object detectors (Version 1). arXiv.

[20] Ariza-Sentís M., Vélez S., Martínez-Peña R., Baja H., Valente J. (2024). Object detection and tracking in Precision Farming: A systematic review. Comput. Electron. Agric..

[21] Zhang X., Li H., Meng F., Song Z., Xu L. (2022). Segmenting Beyond the Bounding Box for Instance Segmentation. IEEE Trans. Circuits Syst. Video Technol..

[22] Sheng X., Kang C., Zheng J., Lyu C. (2023). An edge-guided method to fruit segmentation in complex environments. Comput. Electron. Agric..

[23] Sapkota R., Karkee M. (2024). Comparing YOLOv11 and YOLOv8 for instance segmentation of occluded and non-occluded immature green fruits in complex orchard environment (Version 3). arXiv.

[24] Sapkota R., Meng Z., Churuvija M., Du X., Ma Z., Karkee M. (2026). Comprehensive performance evaluation of YOLOv12, YOLO11, YOLOv10, YOLOv9 and YOLOv8 on detecting and counting fruitlet in complex orchard environments. Agric. Commun..

[25] Ratprakhon K., Neubauer W., Riehn K., Fritsche J., Rohn S. (2020). Developing an Automatic Color Determination Procedure for the Quality Assessment of Mangos (*Mangifera indica*) Using a CCD Camera and Color Standards. Foods.

[26] Furqan Mhd Ikhsan M., Dalimunthe A. (2021). Detection of Ripeness of Manggosteen Fruit Using Hsv Color Space Trans-formation Method. Int. J. Inf. Syst. Technol..

[27] Kirillov A., Mintun E., Ravi N., Mao H., Rolland C., Gustafson L., Xiao T., Whitehead S., Berg A.C., Lo W.-Y. (2023). Segment Anything (Version 1). arXiv.

[28] Zhang A.-A., Shu C., Xie L., Wang Q.-H., Xu M.-Q., Pan Y., Hao W.-L., Zheng Z.-A., Jiang Y.-H., Xiao H.-W. (2025). Enhancing shelf-life of dried goji berry: Effects of drying methods and packaging conditions on browning evolution. Food Res. Int..

[29] Ma C., Liu F., Ran L., Mi J., Lu L., Wang S., Ge X., Jin B., Zhang L., Yan Y. (2025). Assessment of Phenotypic Characteristics, Polysaccharide Composition, and Hypoglycemic Potential in Different Commercial Grades of *Lycium barbarum*: A Comprehensive Study Using HPLC and NMR. Foods.

[30] Fan P., Lang G., Guo P., Liu Z., Yang F., Yan B., Lei X. (2021). Multi-Feature Patch-Based Segmentation Technique in the Gray-Centered RGB Color Space for Improved Apple Target Recognition. Agriculture.

[31] Li M., Xiong J., Deng R., Yao T., Tyree R.N., Hiremath G., Huo Y. (2024). Automatic Image Unfolding and Stitching Framework for Esophageal Lining Video Based on Density-Weighted Feature Matching (Version 1). arXiv.

[32] Liu S., Zeng X., Whitty M. (2020). A vision-based robust grape berry counting algorithm for fast calibration-free bunch weight estimation in the field. Comput. Electron. Agric..

[33] Yi J., Jiang H., Wang X., Tan Y. (2024). A Comprehensive Review on Sparse Representation and Compressed Perception in Optical Image Reconstruction. Arch. Comput. Methods Eng..

[34] Mirzaei B., Nezamabadi-pour H., Raoof A., Derakhshani R. (2023). Small Object Detection and Tracking: A Comprehensive Review. Sensors.

